# A phase II study of Osimertinib for patients with radiotherapy-naïve CNS metastasis of non-small cell lung cancer: treatment rationale and protocol design of the OCEAN study (LOGIK 1603/WJOG 9116L)

**DOI:** 10.1186/s12885-020-06874-6

**Published:** 2020-05-01

**Authors:** Kazushige Wakuda, Hiroyuki Yamaguchi, Hirotsugu Kenmotsu, Minoru Fukuda, Masafumi Takeshita, Takayuki Suetsugu, Keisuke Kirita, Noriyuki Ebi, Osamu Hataji, Satoru Miura, Kenji Chibana, Isamu Okamoto, Kenichi Yoshimura, Kazuhiko Nakagawa, Nobuyuki Yamamoto, Kenji Sugio

**Affiliations:** 1 Division of Thoracic Oncology, Shizuoka Cancer Center Hospital, 1007 Shimonagakubo Nagaizumi-cho Suntou-gun, Shizuoka, 411-8777 Japan; 2 Department of Respiratory Medicine, Nagasaki University Graduate School of Biomedical Sciences, 1-7-1 Sakamoto, Nagasaki, 852-8501 Japan; 3 Department of Respiratory Medicine, Nagasaki University Graduate School of Biomedical Sciences and Clinical Oncology Center, Nagasaki University Hospital, 1-7-1 Sakamoto, Nagasaki, 852-8501 Japan; 4 Department of Respiratory Medicine, Kitakyushu Municipal Medical Center, 2-1-1 Bashaku, Kokurakita-ku, Kitakyushu, Fukuoka, 802-0077 Japan; 5 Department of Respiratory Medicine, Sendai Medical Association Hospital, 4107-7 Nagatoshi-cho, Satsumasendai, Kagoshima, 895-0005 Japan; 6 Department of Thoracic Oncology, National Cancer Center Hospital East, 6-5-1 Kashiwanoha, Kashiwa, Chiba, 277-8577 Japan; 7 Department of Respiratory Medicine, Iizuka Hospital, 3-83 Yoshiomachi, Iizuka, Fukuoka, 820-8505 Japan; 8 Department of Respiratory Medicine, Matsusaka Municipal Hospital Respiratory Center, 1550 Tonomachi, Matsusaka, Mie 515-8544 Japan; 9 Department of Internal Medicine, Niigata Cancer Center Hospital, 2-15-3 Kawagishi-cho, Chuo-ku, Niigata, 951-8566 Japan; 10 Department of Respiratory Medicine, National Hospital Organization, Okinawa National Hospital, 3-20-14 Ganeko, Ginowan, Okinawa, 901-2214 Japan; 11 Research Institute for Diseases of the Chest, Graduate School of Medical Sciences, Kyushu University, 3-1-1 Maidashi, Higashi-ku, Fukuoka, 812-8582 Japan; 12 Department of Biostatistics, Innovative Clinical Research Center, Kanazawa University Hospital, Takara-machi, Kanazawa, Ishikawa 920-8641 Japan; 13 Department of Medical Oncology, Kindai University Faculty of Medicine, 377-2 Ohno-higashi, Osaka-Sayama, Osaka, 589-8511 Japan; 14 Internal Medicine III, Wakayama Medical University, 811-1 Kimiidera, Wakayama, 641-8509 Japan; 15 Department of Thoracic and Breast Surgery, Oita University Faculty of Medicine, 1-1 Idaigaoka, Hasama-machi, Yufu, Oita 879-5593 Japan

**Keywords:** Non-small cell lung cancer, EGFR T790M, CNS metastasis, Brain metastasis, Osimertinib

## Abstract

**Background:**

Patients with activating epidermal growth factor receptor (*EGFR*) mutations are highly responsive to EGFR-tyrosine kinase inhibitors (TKIs). However, it has been reported that approximately 15–30% of patients treated with EGFR-TKIs experience central nervous system (CNS) progression, and patients with *EGFR* mutations exhibit a higher incidence of brain metastasis than those without such mutations. The efficacy of osimertinib for treating CNS metastasis has been reported, but its efficacy for CNS metastasis in radiotherapy-naïve patients is unclear.

**Methods:**

In the present prospective two-cohort phase II trial, 65 patients (T790M cohort, 40 patients; first-line cohort, 25 patients) with radiotherapy-naïve CNS metastasis of *EGFR* mutation-positive non-small cell lung cancer (NSCLC) will be included. Patients will be treated once-daily with osimertinib 80 mg. The primary endpoint is the response rate of brain metastasis as assessed using the PAREXEL criteria. Key secondary endpoints are progression-free survival and the response rate of brain metastasis as assessed using the RECIST criteria. We will exploratorily analyze the relationships of the blood concentration of osimertinib with its efficacy against brain metastasis of NSCLC and the accumulation of osimertinib in cerebrospinal fluid and evaluate tumor-derived DNA from plasma specimens for mutations in *EGFR* and other genes. Recruitment, which in October 2016, is ongoing.

**Discussion:**

Although previous reports revealed the efficacy of osimertinib for CNS metastasis, these reports only involved subgroup analysis, and the efficacy of osimertinib for patients with previously untreated CNS metastasis remains unclear. The OCEAN study is the only trial of osimertinib for patients with untreated brain metastasis of NSCLC. This study should provide novel data about osimertinib. If the results of the OCEAN study are positive, then avoidance of radiotherapy will be recommended to patients harboring *EGFR* mutations and brain metastasis.

**Trial registration:**

UMIN identifier: UMIN000024218 (date of initial registration: 29 September 2016). jRCT identifier: jRCTs071180017 (date of initial registration: 13 February 2019).

## Background

Patients with activating epidermal growth factor receptor (*EGFR*) mutations are highly responsive to EGFR-tyrosine kinase inhibitors (TKIs). However, it has been reported that approximately 15–30% of patients treated with EGFR-TKIs experience central nervous system (CNS) progression, and patients with *EGFR* mutations exhibit a higher incidence of brain metastasis than those without such mutations [1–3]. Although radiotherapy (RT), such as whole-brain radiotherapy (WBRT) and stereotactic radiotherapy, is a standard treatment for CNS metastasis, the median survival time of patients receiving WBRT is only 4–8 months [4, 5]. It has also been reported that the risk of cognitive dysfunction was increased by WBRT. Thus, a need exists for new treatment strategies other than RT.

A majority of patients treated with EGFR-TKIs experience disease progression after 10–12 months, and approximately 50% of patients develop acquired resistance caused by the *EGFR* T790M mutation [6]. Osimertinib is an irreversible EGFR-TKI that selectively inhibits both EGFR-TKI–sensitizing mutations and the *EGFR* T790M mutation. The results of the AURA3 trial, a phase III trial comparing osimertinib with platinum and pemetrexed for patients with non-small cell lung cancer (NSCLC) harboring the *EGFR* T790M mutation who were previously treated with EGFR-TKIs, were reported in 2017 [7]. Osimertinib significantly prolonged progression-free survival (PFS) compared with the effects of platinum and pemetrexed (median PFS: 10.1 months versus 4.4 months, *p* < 0.001), and it has emerged as the standard treatment for patients with NSCLC harboring the *EGFR* T790M mutation who experience disease progression during or after treatment with EGFR-TKIs. In 2018, the FLAURA trial, a phase III trial comparing osimertinib with gefitinib or erlotinib in the first-line setting for patients with *EGFR* mutation who had not previously received EGFR-TKIs, was reported [8]. In the study, PFS was significantly longer in the osimertinib arm than in the gefitinib/erlotinib arm (median PFS: 18.9 months versus 10.2 months, p < 0.001). Osimertinib is currently used in the first-line setting for patients harboring *EGFR*-sensitive mutations.

It was reported that osimertinib displayed greater penetration into the brain than rociletinib or gefitinib in a preclinical model [9]. Osimertinib is expected to have efficacy in patients with CNS metastasis; indeed, a subgroup analysis of patients with CNS metastasis has been reported [10]. In that report, pooled data from two phase II trials of osimertinib in the treatment of patients with NSCLC harboring the *EGFR* T790M mutation (AURA extension and AURA2) were analyzed. Of the 411 patients who participated in these trials, 128 had CNS metastasis, and 50 had one or more measurable CNS lesions. The rate of confirmed CNS responses to osimertinib was 54% in patients with measurable CNS metastasis. Nineteen patients with brain metastasis had already been treated with RT within 6 months before the first dose, and the CNS response in this subgroup was 32%. Conversely, in 31 patients who received RT more than 6 months before the first drug dose or who did not receive RT, the rate of CNS responses to osimertinib was 68%. However, in a subgroup analysis of AURA 3, the CNS response rate of osimertinib for patients who were treated with RT within 6 months before randomization was 64% [11]. Contrarily, the CNS response for patients who did not receive RT within 6 months before randomization was 34%. In these two subgroup analyses, the efficacy of osimertinib for CNS metastasis in patients who did not receive RT was controversial. Because it was assumed that RT might have influenced the efficacy of osimertinib in these studies, the efficacy of osimertinib for CNS metastasis in patients who did not previously receive RT is unclear. A prior multi-institutional retrospective analysis found that patients with brain metastasis who underwent stereotactic radiosurgery (SRS) before EGFR-TKI therapy exhibited better overall survival (OS) than their counterparts who received EGFR-TKIs before SRS [12]. The efficacy of osimertinib for brain metastasis in patients who did not receive RT is controversial, and it is unclear whether EGFR-TKIs should be administered without brain RT to such patients. Thus, a trial assessing the efficacy of osimertinib for patients with untreated CNS metastasis is needed.

## Methods

### Study design

**Fig. 1:**
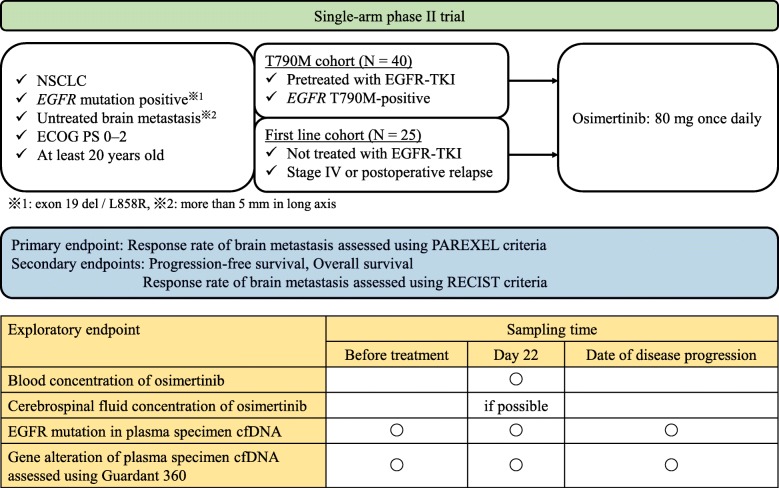
Study schema

The OCEAN study is a multicenter, single-arm phase II study. The overall objective is to evaluate the efficacy of osimertinib for untreated CNS metastasis. Figure 1 provides an overview of the OCEAN study scheme. Patients will orally receive osimertinib at 80 mg once daily until progression, death, or withdrawal of consent to participate in this study.

Before registration in this trial, contrast-enhanced computed tomography (CT) of the chest and abdomen and contrast-enhanced magnetic resonance imaging (MRI) of the brain with a slice thickness of less than 3 mm are required. CT and MRI will be performed every 6 weeks in the first year after the date of registration and every 3 months thereafter.

The OCEAN study is being conducted in compliance with the principles of the Declaration of Helsinki, and it was approved by the central review board of Clinical Research Network Fukuoka. This trial is registered in the University Hospital Medical Information Network Trials Registry (UMIN000024218) and Japan Registry of Clinical Trials (jRCTs071180017).

### Eligibility criteria

The main patient inclusion and exclusion criteria are shown in Table 1. Initially, the OCEAN study aimed to include only patients with NSCLC harboring the *EGFR* T790M mutation who experienced disease progression during or after treatment with EGFR-TKIs. However, patient recruitment was slow because osimertinib has been approved for use in the first-line setting. In addition, it is also important to assess the efficacy of osimertinib for untreated CNS metastasis in EGFR-TKI–naïve patients. We amended the study protocol and established a first-line cohort including previously untreated patients harboring *EGFR*-sensitive mutations regardless of the presence of the *EGFR* T790M mutation.

**Table 1:** Key inclusion and exclusion criteria

Key inclusion criteria		
	T790M cohort	First-line cohort
✓	Histologically or cytologically confirmed non-small cell lung cancer	
✓	Confirmed *EGFR* mutations (exon 19 deletion, exon 21 L858R point mutation)	
✓	Radiological disease progression following first- or second-generation EGFR-TKIs	Previously untreated with EGFR-TKIs
✓	Confirmed *EGFR* T790M mutation detected from tumor or plasma sample after disease progression from prior treatment	
✓		Stage IV or postoperative relapse
✓	Patients must have a brain metastasis lesion of 5 mm or more in size in the long axis irrespective of the presence of extracranial metastases Patients with brain metastasis requiring emergent therapy are excluded	
✓	No prior radiation therapy for brain metastasis	
✓	Patients aged at least 20 years at the time of informed consent	
✓	ECOG performance status 0–2	
✓	Adequate organ function	
✓	Mean corrected QT interval not exceeding 471 ms	
✓	Written informed consent obtained from the patient	
Key exclusion criteria		
✓	Symptomatic brain metastasis requiring radiation therapy or surgical resection	
✓	Severe complications	
✓	Presence of active double cancers (synchronous cancers and metachronous cancers with a disease-free interval of no more than 5 years)	
✓	Prior treatment with anti-PD-1, anti-PD-L1, anti-CD137, and anti-CTLA-4 antibody	
✓	Pregnancy or planned or expected pregnancy	
✓	Lactation in women	
✓	History of interstitial lung disease, drug-induced interstitial lung disease, and radiation pneumonitis requiring steroid treatment	
✓	Presence of symptomatic superior vena cava syndrome	
✓	Presence of psychiatric disorder or mental symptoms	
✓	History of hypersensitivity to osimertinib and any excipients of osimertinib	

*Abbreviations*: *EGFR* Epidermal growth factor receptor, *TKI* Tyrosine kinase inhibitor, *ECOG* Eastern Cooperative Oncology Group, *PD-1* Programmed cell death-1, *PD-L1* Programmed cell death-ligand 1, *CD* Cluster designation, *CTLA-4* Cytotoxic T-lymphocyte antigen-4

### Study endpoints

The initial primary endpoints of the OCEAN study at the start of study enrollment were the response rate of brain metastasis (BMRR) as assessed using the PAREXEL criteria and PFS (https://www.parexel.com/files/5214/0422/3830/MI_Brain_Metastases_White_Paper_JUN_14.pdf). We established the first-line cohort to assess the efficacy of osimertinib for untreated CNS metastasis in EGFR-TKI–naïve patients, and thus, PFS was changed to a secondary endpoint. The PAREXEL criteria represent a tool for assessing brain metastasis, and they have recently been used in several trials. In these criteria, the target lesion of brain metastasis has a size of 5 mm or more in the long axis. Some reports indicated that the size of brain metastases was significantly smaller for patients with mutant *EGFR* than in those with wild-type *EGFR*, and we believe that the PAREXEL criteria are also useful for evaluating evaluate small brain metastases [3, 13]. A maximum of five lesions in the brain will be chosen, and the sum of their diameters will be calculated (sum of the longest axes of all target brain lesions). Non-target lesions include all measurable lesions not chosen as target lesions and lesions with a long axis of < 5 mm. There will be no limit on the number of non-target lesions. The assessment of brain metastasis response is similar to that using the RECIST criteria [14]. The response of each target lesion will be classified as complete response (CR), partial response (PR), stable disease (SD), progressive disease (PD), or not evaluable (NE). The response of each non-target lesion will be classified as CR, non-CR/non-PD, PD, or NE. Key secondary endpoints of the OCEAN study include PFS, the overall response rate (ORR) as assessed using the RECIST criteria, BMRR as assessed using the RECIST criteria, brain metastasis-related PFS, OS, BMRR in the first-line cohort, and PFS in the first-line cohort. PFS is defined as the time from the date of registration to that of death or disease progression, whichever occurs first. Brain metastasis-related PFS is defined as the time from the date of registration to that of death or brain metastasis progression, whichever occurs first. OS is defined as the time from the date of registration to that of death.

To investigate the relationship between the concentration of osimertinib and the treatment effect, we are exploratorily assessing the blood concentration of osimertinib at day 22, which considered to represent steady state [15]. We are also determining the cerebrospinal fluid concentration of osimertinib to analyze its penetration into this fluid. It is also necessary to measure its blood concentration. Blood specimens will thus be collected once 22 days after osimertinib administration. Cerebrospinal fluid is collected on a voluntary basis. The blood and cerebrospinal fluid concentrations of osimertinib are being assessed using HB-13-050 and HB-13-081 (HPLC-MS/MS). To determine whether the *EGFR* C797S mutation is present before progression, we are also evaluating tumor-derived DNA for *EGFR* mutations, including the C797S point mutation, in plasma specimens. Plasma specimens for *EGFR* mutation analysis are collected three times: before treatment, 22 days after the administration of osimertinib, and on the date of diagnosis of progressive disease. In the first-line cohort, we will also evaluate tumor-derived DNA by the Guardant 360 liquid biopsy for gene alterations in plasma specimens to simultaneously analyze the mechanism of acquired resistance to osimertinib and the *EGFR* mutation status. Guardant 360 assesses point mutations (SNVs) and deletion variants (Indels) in 74 genes, amplification in 18 genes, and fusions in 6 genes. The cut-off for mutant variants is ≥0.04% for SNVs, Indels, and fusions and ≥ 2.18 copies for amplifications.

### Statistical considerations

The primary endpoint of the OCEAN study is BMRR as assessed using the PAREXEL criteria in the full analysis set population, excluding the first-line cohort. To estimate BMRR, we refer to the results of the AURA trial [16]. In this trial, ORR was 61% (95% confidence interval [CI] = 52–70%). We will consider osimertinib effective if similar efficacy is observed in patients with untreated brain metastasis. On the basis of the lower limit of the 95% CI of ORR in the AURA trial, we set a threshold value of BMRR of 50%. We also set an expected value of 70% based on the upper limit of the 95% CI of ORR in the AURA trial. First, based on one-sided alpha = 0.05 and power = 0.9, the sample size for the OCEAN study was calculated to be 60, considering the possibility of patient withdrawal. However, we amended our statistical hypothesis from power = 0.9 to 0.8 because of the slow accrual rate. The sample size was changed to 37, and the amended sample size was calculated to be 40 considering dropouts. Considering the threshold and expected values of BMRR of 55 and 80%, respectively, in the first-line cohort, the sample size of the first-line cohort was calculated to be 25 considering dropouts at one-sided alpha = 0.05 and power = 0.8.

## Discussion

Although previous reports described the efficacy of osimertinib for treating CNS metastasis, these studies only involved subgroup analysis, and the efficacy of osimertinib for patients with previously untreated CNS metastasis remained unclear. The OCEAN study has several limitations. First, whether brain metastases had the EGFR T790M mutation was unclear because EGFR T790M was assessed using extracranial tissue or plasma samples. However, CSF sampling is only performed in patients with suspected meningeal carcinomatosis prior to osimertinib in clinical practice; therefore, requiring CSF sampling before enrollment would be difficult. We believe that the OCEAN study is valuable because its study design is suitable for clinical practice. Second, the OCEAN study was a single-arm phase II study. Patients treated with radiotherapy prior to osimertinib might have experience better treatment efficacy than those treated with osimertinib prior to radiotherapy. Magnuson et al. performed a retrospective analysis and found that upfront EGFR-TKI therapy and deferral of radiotherapy was associated with inferior OS in patients with NSCLC harboring EGFR mutations and CNS metastasis [12]. However, patients who were not treated with EGFR-TKI after radiotherapy were excluded. Because radiotherapy might prevent osimertinib, which has been shown to be effective for patients with EGFR mutations, and because the risk of cognitive dysfunction is increased by WBRT, we considered that comparison of osimertinib and radiotherapy in patients with CNS metastasis would be difficult. To our knowledge, the OCEAN study is the only trial of osimertinib for patients with untreated brain metastasis that has been initiated to date. This study should provide novel data about osimertinib. If the results of this study meet the primary endpoint, then it will be recommended that patients with brain metastasis harboring *EGFR* mutations forgo RT.

## Data Availability

Not applicable.

## Abbreviations

BMRR: Response rate of brain metastasis
CNS: Central nervous system
CT: Computed tomography
EGFR: Epidermal growth factor receptor
MRI: Magnetic resonance imaging
NSCLC: Non-small cell lung cancer
ORR: Overall response rate
OS: Overall survival
PFS: Progression-free survival
RT: Radiation therapy
SRS: Stereotactic radiosurgery
TKI: Tyrosine kinase inhibitors
WBRT: Whole-brain radiation therapy
